# The personal and national costs of lost labour force participation due to arthritis: an economic study

**DOI:** 10.1186/1471-2458-13-188

**Published:** 2013-03-03

**Authors:** Deborah J Schofield, Rupendra N Shrestha, Richard Percival, Megan E Passey, Emily J Callander, Simon J Kelly

**Affiliations:** 1 NHMRC Clinical Trials Centre, University of Sydney, Sydney, Australia; 2 Sydney School of Public Health, University of Sydney, Sydney, Australia; 3 National Centre for Social and Economic Modelling, University of Canberra, Canberra, Australia; 4 University Department of Rural Health (North Coast), University of Sydney, Lismore, Australia

**Keywords:** Economic impacts, Income, Taxation, GDP, Arthritis, Retirement

## Abstract

**Background:**

The costs of arthritis to the individuals and the state are considerable.

**Methods:**

Cross-sectional analysis of the base population of Health&WealthMOD, a microsimulation model of 45 to 64 year old Australians built on data from the Australian Bureau of Statistics’ Survey of Disability, Ageing and Carers and STINMOD, an income and savings microsimulation model.

**Results:**

Individuals aged 45 to 64 years who had retired early due to arthritis had a median value of AU$260 in total weekly income whereas those who were employed full time were likely to average more than five times this. The large national aggregate impact of early retirement due to arthritis includes AU$9.4 billion in lost GDP, attributable to arthritis through its impact on labour force participation.

When looking at the ongoing impact of being out of the labour force those who retired from the labour force early due to arthritis were estimated to have a median value of total savings by the time they are 65 of as little as $300 (for males aged 45–54). This is far lower than the median value of savings for those males aged 45–54 who remained in the labour force full time, who would have an estimated $339 100 of savings at age 65.

**Conclusions:**

The costs of arthritis to the individuals and the state are considerable. The impacts on the state include loss of productivity from reduced workforce participation, lost income taxation revenue, and increased government support payments – in addition to direct health care costs. Individuals bear the economic costs of lost income and the reduction of their savings over the long term.

## Background

Arthritis is a common and costly health condition internationally [1,2]. The direct medical costs of arthritis are significant, with the condition being the fourth most common reason for seeking general practitioner medical care [3]. Treatment of arthritis in Australia cost around $4 billion (AU) in health system expenditure in 2004–05, the fourth largest cause of health expenditure in the country [4]. However, this figure covers only the direct medical costs, and the indirect costs, are considered to be larger [5,6].

Within Australia arthritis has been listed as a ‘National Health Priority Area’ [7], and affects 15% of the population [3]. By 2020 the prevalence of the condition is estimated to increase, with arthritis potentially affecting 35% of the Australian population [6]. Arthritis can cause significant activity limitation, and is responsible for around 13% of the disability reported in Australia [3,4].

Due to its impact on functional ability, arthritis is associated with decreased labour force participation rates [8]. Within Australia the impact of arthritis on labour force participation among people in the pre-retirement age group of 45 to 64 years is significant – with people suffering from arthritis being 3 times more likely to be out of the labour force than those with no chronic health condition [9]. It is also the condition responsible for the second highest number of older workers being out of the labour force [9]. Workforce absence comprises a significant proportion of the burden of the disease in numerous other countries, including New Zealand, Canada and the United States [5,10-12].

With the ageing of the global population, the 45 to 64 year age group is making up an increasing proportion of the working population and hence their workforce participation is vital for economies internationally [13-15]. As the prevalence of arthritis increases with age [3], arthritis may be a growing source of workforce absence in this age group. Of those who are aged 45 to 64 years within Australia who identify arthritis as their main health condition, 50% are not in the labour force [9], making early retirement due to arthritis a serious issue. Early retirement is likely to reduce the income and wealth available to the individual, and also place a burden on government due to the lost income taxation revenue and the increase in government benefit payments to the retired individuals.

There have been no detailed studies on the individual impacts of early retirement due to arthritis. The studies that have looked at the indirect costs of the condition have generally focused only on loss of employment income and most exclude, for example, reductions in income from other sources, reductions in taxation revenue from earned income and the reduction in wealth over the longer term. In addition, previous research has not examined disaggregated individual outcomes but rather only aggregated outcomes.

This paper quantifies how much reduced income, reduced taxation revenue, increased benefits payments and lost GDP are attributable to arthritis when it leads to the early retirement of 45 to 64 year old Australians. The paper will also estimate the extent to which those who exit the workforce early due to arthritis have less savings by the time they reach the traditional retirement age of 65. This will give a more complete picture of the costs of arthritis, and show how much could be saved if the disability from arthritis had been prevented through primary prevention or improved treatment and individuals remained in the labour force.

## Methods

### Building the microsimulation model: health&WealthMOD

The output dataset of a microsimulation model, Health&WealthMOD, which is Australia’s first microsimulation model of income, wealth, health and disability, was used to analyse the impacts that arthritis has on labour force participation, personal income and wealth, and government revenue and expenditure amongst Australians aged 45 to 64 years. The process by which Health&WealthMOD was built is described in detail in Schofield *et al*. [16] and will be outlined below.

The base population of Health&WealthMOD was unit record data extracted from the Survey of Disability, Ageing and Carers conducted by the Australian Bureau of Statistics in 2003 [17]. From this dataset, individual records were extracted for those aged 45–64 years. The 2003 SDAC covered both private and non-private dwellings, excluding gaols and correctional institutions. The ABS selected households at random using multistage sampling techniques and surveyed every individual within the household. There were 36 241 respondents in 14 019 households, and 5 145 individuals from 303 non private dwellings and 564 care-accommodation establishments. The response rate for those in private and non private dwellings was 89%, and for those in care-accommodation 90% (Australian Bureau of Statistics 2004).

The details extracted from the Survey of Disability, Ageing and Carers for each individual in the base population included demographic variables (for example, age, sex, family type, state of residence, and ethnic background), socioeconomic variables (level and field of education, income decile, type of benefit received), labour force variables (labour force participation, employment restrictions, retirement), and health and disability variables (chronic conditions, health status, type and extent of disability, support and care required). This information formed the ‘base population’ of Health&WealthMOD.

Respondents in the Survey of Disability, Ageing and Carers reported what their main health conditions were and their responses were classified by the Australian Bureau of Statistics using ICD10 codes. For this study people who reported their main health condition as “arthritis and related disorders” (ICD10 code M00-19) were identified. In the Survey of Disability, Ageing and Carers, if respondents were not in the labour force, their stated reason for this was recorded. In this study those who stated they were out of the labour force due to their illness and listed arthritis as their main condition were considered to be out of the labour force due to arthritis.

Using a separate microsimulation model—STINMOD— more detailed economic information such as continuous individual income, amount of government support payments received, tax liability and value of wealth was imputed onto the base population of Health&WealthMOD. STINMOD is Australia’s leading model of income tax and government support payments [18,19], and is maintained and developed for the Australian Government by the National Centre for Social and Economic Modelling.

Income information from STINMOD was imputed onto the base population of Health&WealthMOD by identifying persons with similar characteristics on STINMOD and “donating” their income and wealth information onto Health&WealthMOD using a process commonly used in microsimulation modelling called synthetic matching [20]. Nine variables: sex (2 groups), income unit type (4 groups), type of government pension/support (3 groups), income quintile (5 groups), age group (4 groups), labour force status (4 groups), hours worked per week (5 groups), highest educational qualification (2 groups) and home ownership (2 groups), that were common to both datasets and strongly related to income were chosen as matching variables for synthetic matching. Using these variables, over 99 per cent of records were able to be exactly matched between the two datasets for all characteristics (labour force status, income unit type, type of government pension/support, income quintile, five age groups, sex, hours worked per week, highest educational qualification and home ownership), with the exception of age group where 94% of records were matched exactly [16]. Priority was given to those variables that were the strongest predictors of income in the matching algorithm.

The data were then aged to reflect the 2009 Australian 45 to 64 year old population. The up-rating was used to account for the disability and illness, demographic, labour force, earnings growth and other changes that had occurred between 2003 and 2009.

### Estimating savings at age 65

*Health&WealthMOD* then used microsimulation techniques to project the financial circumstances of each person in the base data when they would be aged 65 years, the traditional retirement age. For the comparison in this study, it was assumed that persons not in the labour force due to arthritis would remain out of the labour force and those individuals who were participating in the labour force would continue participating until age 65 years. As there was no available data from which to project the growth in debts for this specific population, it was assumed that there would be no change in the real value of the debt.

For the purposes of this study, estimates of the level of retirement income that could be obtained by converting superannuation [1] and other savings into an income stream at age 65 were modelled. To estimate savings and income for each person to the age of 65 years, respondents were assumed to continue earning at the same level, with an adjustment to increase earnings in line with long term average earnings growth rate less inflation (the real earnings growth rate). This rate was estimated by the change in Average Weekly Ordinary Times Earnings (AWOTE) trend data between May 1989 and May 2009 [21] and inflation as measured by the change in the Consumer Price Index (CPI) between June 1989 and June 2009 [22]. The real earnings growth rate was estimated at 1.60 per cent per annum using this calculation.

In this paper, household net worth excluding the value of equity in the family home was used as an indicator of income producing assets at age 65.

#### Superannuation

All superannuation was assumed to be invested in an accumulation fund. Accumulation funds operate like a bank account with personal accounts and the balance being a function of contributions and investment returns. To grow this fund balance to age 65 years, the occurrence and level of voluntary contributions were modelled based on a person’s age and sex. The probability of making a voluntary contribution increases with age and males were more likely to make a contribution than females. The level of voluntary contribution was a percentage of earnings and increased with age. The detailed description of the derivation of these probabilities can be found in Harding *et al*. 2009 [23].

#### Asset growth

The growth rates used for housing and shares were the compound growth rate over the last 20 years from the Housing index (based on ABS House Price Index for eight capital cities Tables one and nine from June 1989 to June 2009 [24]), 5.97%; and the Australian Stock exchange All Ordinaries Index June 1989 – June 2009 [25], 4.88%.

For cash deposits, two different rates were used. The low cash rate was applied to cash deposits below $5 000 and the high cash rate was applied to deposits above this level. The low rate used was an average interest rate paid by banks on a transaction account of $5000 over the last ten years [26]. The average was 0·03 per cent. For cash deposits over $5 000, the same data source provided the average interest rate on a 3-month bank term deposit of $10 000. This produced an average return of 3·8 per cent over the last ten years.

The superannuation return used in the model is the net return for a balanced fund (60% to 75% invested in the share market and the remainder in low risk investments) over the 10 years to June 2009 as measured by superratings.com.au (SR50 Balanced (60–76) Index. This gave an average annual nominal return of 5·2 per cent. The 20-year average consumer price index, 2.85 per cent was used as an indicator of inflation and subtracted from the average growth rates to give real growth rates.

#### Savings

The focus of the *Health&WealthMOD* simulation is to estimate the level of retirement savings that will be available to an individual at age 65. This estimate includes savings that a person makes from their income each year, and changes in the values of asset owned. The latter of these was calculated based on the value of the asset and the investment return assigned to that type of asset.

Long term trend analysis of HILDA [27] panel data was used to estimate rates of personal additional savings, which found that the level of personal additional savings when expressed as a percentage of disposable income were close to zero (median equals −0·5 per cent). Based on this analysis, it was decided to assign an additional savings rate of zero to each person for this paper.

#### Annuity calculation

To estimate the income from savings in retirement, it was assumed that respondents would convert all of their superannuation, cash, shares, and other properties into cash and invest it in an investment vehicle such as a lifetime annuity. The income stream is designed to be drawn down evenly over the duration of their life expectancy. Life expectancy is derived from the Australian Life Tables for a person aged 65 in 2007 [28]. The life expectancy of a male aged 65 in 2007 is 18·5 years and a female of this age is 21·6 years. This means that a female will have withdrawn all of their savings (including interest) by the age of 87 (65 plus 22 years) and males by age of 84 years (65 plus 19 years).

### GDP estimation

For the calculation of GDP lost due to people being out of the labour force due to ill health the following equations were utilised, with the values being publically available through the ABS:

$$\text{GDP}\;\text{without}\;\text{missing}\;\text{workers}=\left(\text{GDP}/H\right)\;x\;\left(H/\text{EMP}\right)x\left(\text{EMP}/\text{LF}\right)\times \left(\text{LF}/{\text{Pop}15}^{+}\right){\times \;\text{Pop}15}^{+}$$

$$\text{GDP}\;\text{with}\;\text{missing}\;\text{workers}=\left(\text{GDP}/H\right)\;x\;\left[H/\left(\text{EMP}+\text{ARTH}\right)\right]\times \left[\left(\text{EMP}+\text{ARTH}\right)/\text{LF}\right]\times \left(\text{LF}/{\text{Pop}15}^{+}\right){\times \;\text{Pop}15}^{+}$$

where GDP = Gross Domestic Product; H = total hours worked; EMP = total number of persons employed; ARTH = total number of people not in the labour force due to arthritis LF=total labour force; and Pop15^+^ = population aged 15 years and over.

This calculation is based on the Commonwealth Treasury’s GDP formula [29], with the input values for GDP, EMP, LF and Pop15+ being publically available through the Australian Bureau of Statistics.

### Statistical methods

Initial descriptive analysis was undertaken to assess the characteristics of those with arthritis who were employed and those who were not in the labour force, specifically looking at the labour force status of those with arthritis and varying numbers of co-morbidities.

Further descriptive analysis was undertaken to assess the difference in labour force determine the mean and median weekly income, taxation payments, and social security benefits attributable to individuals employed full time, employed part time, employed full time with arthritis, employed part time with arthritis and not in the labour force due to arthritis.

A multiple linear regression model of the log of weekly income was used to analyse the percentage differences between the value of weekly income. Results were back transformed by taking the exponentiation to present the estimates in natural terms, the smearing co-efficient developed by Duan [30] was used in this back transformation. Analyses were repeated for weekly transfer income and weekly tax liability. Co-variates: age group, sex, highest education and number of health conditions were adjusted for in all regression models. Regression analysis was undertaken on log-transformed data in order to satisfy the assumptions of linear regression analysis, and regression diagnostics confirmed that the assumptions were reasonably satisfied.

Multiple linear regression models of the log of the value of savings at age 65 and annuity by age 65 were used to analyse the differences between the savings and the annuity of people working full-time with no chronic condition, persons working part-time with no chronic condition, and people not in the labour force due to arthritis. Full-time work with no chronic condition was used as the reference group. Four different classes of wealth were included in total savings – cash, shares, super, investment properties, were analysed in this paper. Multiple regression analyses were undertaken on log-transformed data in order to satisfy the assumptions of linear regression analysis, and regression diagnostics confirmed that the assumptions were reasonably satisfied.

In order to estimate the results for the entire Australian population of the 45–64 years age group, we performed weighted analysis using weights that represented the number of individuals in the Australian population. The analyses were undertaken using SAS V9.1 (SAS Institute Inc., Cary, NC, USA). All statistical tests were two sided with the significance level set at 5%. This research was carried out in compliance with the Helsinki Declaration (http://www.wma.net/en/30publications/10policies/b3/index.html), and the use of the data was approved by Australian Bureau of Statistics.

## Results

Amongst those surveyed in the Survey of Disability, Ageing and Carers who were aged between 45 and 64 years, there were 2 285 who were employed full time with no chronic health condition, 785 who were employed part time with no chronic health condition, 218 who were employed full time with arthritis, and 158 who were employed part time with arthritis, and 111 individuals that were out of the labour force due to arthritis.

Once weighted, these data represented 1, 420, 100 who were employed full time with no chronic health condition, 422 700 who were employed part time with no chronic health condition, 152 600 who were employed full time with arthritis, and 89 300 who were employed part time with arthritis, and 80 900 individuals that were out of the labour force due to arthritis within the Australian population aged 45 to 64 years.

40% of people with arthritis and no other health conditions were employed full time, with a decreasing proportion in full time employment as number of co-morbidities increased. Over half of those with arthritis and one co morbidity were not in the labour force (53%), and 83% of those with arthritis and three of more co morbidities were not in the labour force (Table 1). After adjusting for age, sex and education those with arthritis only had 1.64 times the odds of being out of the labour force compared to those with no health condition (95% CI: 1.13 – 2.38, p=0.0099), and those with arthritis and three of more co morbidities had 8.68 times the odds of being out of the labour force compared to those with no health condition (95% CI: 5.24 – 14.38, p<.0001) (Table 2).

**Table 1:** Employment status of those with various numbers of health conditions, 2009

**Number of conditions**	**Employed FT**	**Employed PT**	**NILF**
No chronic health condition	62%	19%	17%
Arthritis only	41%	19%	39%
Arthritis and one other condition	29%	18%	53%
Arthritis and two other conditions	19%	24%	56%
Arthritis and three or more other conditions	6%	10%	83%

NILF = Not in the labour force.

**Table 2:** Odds ratio of being not in the labour force for those with arthritis compared to those with no chronic health condition, adjusted for age, sex and education, 2009

**Number of conditions**	**OR**	**95% CI**	**p-value**
No chronic health condition	REFERENCE		
Arthritis only	1.64	1.13 – 2.38	0.0099
Arthritis and one other condition	2.95	1.82 – 4.80	<.0001
Arthritis and two other conditions	2.80	1.50 – 5.26	0.0013
Arthritis and three or more other conditions	8.68	5.24 – 14.38	<.0001

Those who were out of the labour force due to arthritis had a median weekly income (including transfer income) of AU$257. This is around half of the median weekly income of those employed part-time with no condition (AU$559 per week), and around one-fifth the median weekly income of those employed full time with no chronic condition - AU$1 226 (Table 3). Of their total weekly income – those not in the labour force due to arthritis received a median value of weekly government transfer income of AU$254, whereas those in employment receive a median value of zero per week. Not being in employment, those out of the labour force due to arthritis pay a median value of zero in tax per week – whereas those employed full-time pay a median value of AU$223 per week in tax.

**Table 3:** Average and median* weekly income, transfer payments and tax liability by labour force status for the Australian population aged 45–64 years, 2009

**Labour force status**	**Weekly income (AU$) received by individuals**			**Weekly transfer income (AU$) received by individuals**			**Weekly tax (includes Medicare levy) (AU$) paid by individuals**		
	**Mean**	**SD**	**Median**	**Mean**	**SD**	**Median**	**Mean**	**SD**	**Median**
Employed full-time, no chronic health condition	1 507	33 575	1 226	9	1082	0	344	11 746	223
Employed part-time, no chronic health condition	657	11 714	559	28	1661	0	78	3 066	30
Employed full-time, with arthritis	1 397	21 427	1 316	6	709	0	310	7 358	234
Employed part-time, with arthritis	577	8 141	494	37	1 939	0	56	2 022	22
Not in labour force due to arthritis	283	4 460	257	226	3 703	254	3	652	0

*all results given in 2009 Australian dollars (AU).

Those employed full time with arthritis received slightly more per week in total income and transfer income than those employed full time with no health condition; whereas those employed part time with arthritis received slightly less in total income and transfer income than those employed part time with no health condition.

When compared to those with no health condition in full time employment and adjusted for age, sex and education, those out of the labour force due to arthritis receive 82 per cent less per week on average in total income (95% CI: -88.3, -71.6, p<.0001) (Table 4). They also pay significantly less per week in taxation (−99.9%, 95% CI:-100.0, -99.9, p<.0001), and receive significantly more in government transfer payments (12 988.2%, 95% CI: 6 577.2, 25 554.6, p<.0001).

**Table 4:** Differences in average weekly income, transfer payments and tax liability between labour force status, adjusted for age group, sex and education, for the Australian population aged 45–64 years, 2009

**Labour force status**	**Income**			**Transfer income**			**Tax liability (includes Medicare levy)**		
	**% difference**	**95% CI**	**p-value**	**% difference**	**95% CI**	**p-value**	**% difference**	**95% CI**	**p-value**
Employed full-time, no chronic health condition		*Reference*			*Reference*			*Reference*	
Employed part-time, no chronic health condition	−55.7	(−61.3, -49.3)	<.0001	73.3	(33.7, 124.6)	<.0001	−90.6	(−93.1, -87.1)	<.0001
Employed full-time, with arthritis	−15.0	(−32.4, 6.9)	0.1640	3.4	(−17.1, 28.8)	0.7698	−15.8	(−43.2, 24.9)	0.3930
Employed part-time, with arthritis	−53.5	(−61.4, -44.0)	<.0001	97.0	(10.7, 250.4)	0.0211	−93.0	(−96.6, -85.8)	<.0001
Not in labour force due to arthritis	−81.8	(−88.3, -71.6)	<.0001	12 988.2	(6 577.2, 25 554.6)	<.0001	−99.9	(−100.0, -99.9)	<.0001

Those employed part-time with no long term health condition and those employed part time with arthritis also had significantly lower incomes (−55.7%, 95% CI: -61.3, -49.3, p<.0001; -53.5, 95% CI: -61.4, -44.0, p<.0001), paid less taxation (−90.6%, 95% CI: -93.1, -87.1, p<.0001; -93.0%, 95% CI: -96.6, -85.8, p<.0001), and received more in transfer payments (73.3%, 95% CI: 33.7, 124.6, p<.0001; 97.0%, 95% CI: 10.7, 250.4, p=0.0211) than those employed full time with no health condition. However, the percentage differences between those employed full time with no health condition and those employed part time, is not as great as those employed full time and those not in the labour force due to arthritis (Table 4).

When aggregated, the national impact of arthritis when it leads to exit from the labour force is AU$3.8 billion in lost income [2] (with reduced private income partially offset by government payments) assuming that otherwise those with arthritis would have had the same labour force participation rates as people with no chronic health conditions (Table 5). The reduction in labour force participation and associated reduction in private earnings resulted in AU$394 million in lost taxation revenue, and an additional AU$291 million in government transfer payments per year.

**Table 5:** National annual impact of persons not in the labour force due to arthritis (adjusted for age, sex and education) for the Australian population aged 45–64 years, 2009

	**Lost Income (million AU$)**	**Additional Transfer Payments (million AU$)**	**Lost Taxation Revenue (million AU$)**
Not in labour force due to arthritis	3,787	290.9	394.0

Note: Based on the differences between persons not in the labour force due to arthritis and the weighted average of persons employed full time and part time with no chronic condition.

As a result of the 80 032 workers missing from the labour force due to early retirement as a result of arthritis, there is a annual loss of AU$9.4 billion in GDP. Total GDP for Australia in 2008–09 was AU$1,263 billion [31].

### Lifetime impacts of being out of the labour force

There was a greater percentage of both males and females who were out of the labour force due to arthritis who would not accumulate any savings by 65 years of age compared to those who remain in the labour force. Depending of age group, 100%, or almost 100%, of individuals who were employed full time would have accumulated some savings at age 65; whereas as little as 83% of those who were out of the labour force due to their arthritis would have done so. Proportionally, more women than men were likely to have no savings at age 65 if they retired from the labour force early due to arthritis (Table 6).

**Table 6:** Comparison of the total savings and annuity at age 65 for people in full time, part time employment, and those who are not in the labour force due to arthritis

			**Total savings (cash, super, shares, other properties)**					**Annuity**		
	**Total population**	**N**	**Total population with savings**	**%**	**Mean ($)**	**Sd**	**Median ($)**	**Mean ($)**	**Sd**	**Median ($)**
**Male, 45-54**										
Employed full time no condition	672 557	**1 142**	669 426	99·5	504 912	622 148	339 121	29 170	35 944	19 592
Employed part time no condition	46 877	**97**	46 877	100·0	329 591	405 851	180 632	19 042	23 447	10 436
Not in labour force due to arthritis	11 016	**8**	10 698	97.1	84 442	120 739	315	4 878	6 975	18
**Male, 55-64**										
Employed full time no condition	278 364	**409**	277 131	99·6	404 659	529 293	251 381	23 378	30 579	14 523
Employed part time no condition	36 626	**70**	36 282	99·1	387 030	588 214	139 439	22 360	33 983	8 056
Not in labour force due to arthritis	16 879	**28**	15 160	89.8	60 253	96 062	7 765	3 481	5 550	449
**Female, 45-54**										
Employed full time no condition	351 870	**584**	349 978	99·5	366 144	370 740	237 496	18 521	18 754	12 014
Employed part time no condition	247 110	**459**	240 029	97·1	238 421	334 096	131 245	12 060	16 900	6 639
Not in labour force due to arthritis	15 306	**26**	14 172	92.6	67 732	113 694	877	3 426	5 751	44
**Female, 55-64**										
Employed full time no condition	113 123	**148**	113 123	100·0	315 735	319 163	214 432	15 971	16 145	10 847
Employed part time no condition	92 095	**159**	91 659	99·5	246 344	306 614	99 455	12 461	15 510	5 031
Not in labour force due to arthritis	37 655	**49**	31 311	83.2	124 530	252 340	32 956	6 299	12 765	1 667

Reflecting the greater proportion of people who were out of the labour force due to ill health having no savings, this group of the population also had far lower median total savings. When savings at age 65 was converted into an annuity at age 65, there was also a corresponding marked reduction in the economic resources available compared to those employed full time or part time and those out of the labour force.

Males aged 45–54 who retired from the labour force early due to arthritis had a median value of total savings by the time they are 65 of only $315. This was far lower than the median value of savings for males in the same age group who remained in the labour force full time, with $339 121 of savings at age 65. The corresponding resultant median annuity available to these groups was $19 592 per annum for those in the labour force full time, and only $18 for those out of the labour force due to their arthritis. Females aged 45–54 who retired due to their arthritis had a median value of savings at age 65 of $877, whereas their counterparts who remained in the workforce full time had $237 496 of savings. The corresponding values of the median annuity for those out of the labour force due to ill health and employed full time was $44, and $12 014 respectively. Males and females in the 55–64 year old age group showed similar differences in accumulated savings by age 65 and available annuity for those employed full time and out of the labour force (Table 6). While the value of total savings at age 65 for those employed part time was consistently less than those employed full time for males and females across both age groups, the value of their savings was still considerably higher than that of those out of the labour force due to arthritis.

The multiple regression models of total savings and annuity show (in Table 7) that, after adjusting for education, those who were out of the labour force due to arthritis had significantly lower savings and annuity at age 65 than those who remained in the labour force full time. Those employed part time also had significantly lower savings and annuity than those employed full time, however the percentage difference was not as great. Males aged 45–54 who were out of the labour force due to their ill health had 98.56% (95% CI: -99.91 to −77.99, p=0.002) less savings and 98.43% (95% CI: -99.89 to −76.60, p=0.003) less annuity at age 65 than their counterparts who remained in the labour force full time. Similarly females aged 45–54 had 99.09% less savings (95% CI: -99.85 to −94.45, p<.0001) and 98.74% less annuity (95% CI: -99.76 to −93.46, p<.0001) at age 65 than females in the same age group in full time employment. Similar differences are reported for males and females in the 55–64 year old age group.

**Table 7:** Percentage difference of total savings and annuity for those working part time or not in the labour force due to arthritis compared to those working full time

	**Total savings (cash, super, shares, other property)**				**Annuity**			
	**% difference**	**p-value**	**95% CI**		**% difference**	**p-value**	**95% CI**	
**Male, 45-54**								
Employed full time no condition	0·00		0·00	0·00	0·00		0·00	0·00
Employed part time no condition	−58.21	0.010	−78.60	−18.41	−59.07	0.009	−78.93	−20.48
Not in labour force due to arthritis	−98.56	0.002	−99.91	−77.99	−98.43	0.003	−99.89	−76.60
**Male, 55-64**								
Employed full time no condition	0·00		0·00	0·00	0·00		0·00	0·00
Employed part time no condition	−45.01	0.039	−68.79	−3.41	−44.23	0.037	−67.74	−3.60
Not in labour force due to arthritis	−97.93	<.0001	−99.54	−90.63	−97.17	<.0001	−99.24	−89.43
	**% difference**	**p-value**	**95% CI**		**% difference**	**p-value**	**95% CI**	
**Female, 45-54**								
Employed full time no condition	0·00		0·00	0·00	0·00		0·00	0·00
Employed part time no condition	−56.28	<.0001	−70.10	−36.06	−53.16	<.0001	−65.61	−36.19
Not in labour force due to arthritis	−99.09	<.0001	−99.85	−94.45	−98.74	<.0001	−99.76	−93.46
**Female, 55-64**								
Employed full time no condition	0·00		0·00	0·00	0·00		0·00	0·00
Employed part time no condition	−51.08	0.001	−67.12	−27.21	−50.04	0.001	−66.14	−26.29
Not in labour force due to arthritis	−97.66	<.0001	−99.58	−86.97	−95.78	<.0001	−98.93	−83.36

## Discussion

Due to increased life expectancies the number of years spent in retirement is increasing, and thus retirees have to be able to finance an increasing period of their lives outside the workforce. As the baby boomers retire, maintaining their preferred living standards will be made even harder due to the high expectations of living standards that have developed [32]. Those who are forced to retire early due to their arthritis will be particular disadvantaged, and may find it difficult to have a decent standard of living.

Another factor which may reduce living standards further is the extent to which people out of the labour force need to draw down their financial resources reducing their already limited savings [33]. Many families are unprepared for the impact of a long-term health condition [34] and it is recognised that in order to cope with the financial burden induced by illness many families will utilise existing savings and sell accumulated assets and capital. Such actions will have negative follow on affects by further depleting asset and capital bases affecting future ability to cope with any financial stress, and leading to fragile financial situations, and reduced income from savings in retirement [35].

In addition to the personal costs of early retirement due to arthritis, the indirect national costs are also considerable. The direct medical costs of arthritis for all ages was $1.4 billion (AU) in 2004–05 in Australia [36], however, the indirect national aggregate impact of early retirement due to arthritis for 45 to 64 year olds was estimated in this paper to be greater. The Australian Institute of Health and Welfare acknowledges that the indirect costs of arthritis “constitute a high financial burden” [37], mostly through loss of labour force participation.

Other studies have also estimated the large indirect costs of arthritis. Access Economics estimated that the cost of lost earnings was almost twice that of the direct health costs in Australia [6], and other studies have estimated that the indirect costs account for up to 80% of the total costs of the condition [38]. The indirect costs are attributed to work disability, work absenteeism and lost earnings [2,10,39-41]. This study improves, methodologically, upon these other studies, which are mostly based on samples that are not nationally representative, and use national average earnings to estimate the cost of workforce absence. This study utilises disaggregated, nationally representative micro-level information on the income and wealth of individuals to calculate the average lost individual income and the long term impact upon wealth. The other studies also do not assess the aggregate national impact of lost earnings, nor do they consider the impact that reduced workforce participation has on governments in terms of lost income taxation and increased government support payments, as this study does.

The reduction in taxation revenue, and increase in the amount of benefits being paid, that is attributable to arthritis will contribute to the strain on government budgets. This comes at a time when there will be added pressure to meet the costs of increased numbers of people receiving disability and aged pensions and the reliance upon health services produced by an ageing population [42,43]. Arthritis, the main long term health condition of 19% of all individuals who have retired early [9], contributes 17% of the total $2.1 billion of taxation revenue lost to governments in 2009 from illness related early retirement, and 19% of the total $1.5 billion paid in government support payments to those retired early due to illness [44].

Managing arthritis early in its development, when there is a rapid decline in workforce participation [45-47], may increase the employment participation rates of people with arthritis. Numerous studies have found that workforce participation of arthritis sufferers can be improved through treatment or workplace modifications [46,48,49]. Investment in preventive health measures is also seen as one way of overcoming the detrimental impacts that ill health has on workforce participation [50]. Studies have found that some arthritis treatments result in increased labour force participation [48,49]. Lacaille *et al.* also found that modifying work-related factors could also increase the labour force participation of arthritis sufferers [46]. Early intervention should be encouraged with the aim of reducing the severity of arthritis in order to prevent the high costs of lost workforce participation to individuals and government.

## Conclusion

The costs of arthritis are considerable both at the individual level and at the aggregate national level. Individuals aged 45 to 64 years who have retired early due to arthritis have 82% lower income then their full time employed counterparts with no chronic health condition - they have an a median value of AU$257 in total weekly income whereas those who are employed full time are likely to average more than five times this. Furthermore, those out of the labour force due to arthritis will accumulate a significantly lower amount of savings by the time they reach the traditional retirement age of 65, and have a correspondingly significantly lower income stream available with them to finance their retirement years.

## Endnotes

^a^Superannuation is the Australian term for private retirement pension plans. Compulsory contributions are made to superannuation by a person’s employer and voluntarily contributions can be made by the employee.

^b^In 2009 Australia dollars – 1 Australian dollar = approx. 0.55GBP in 2009. In 2009 the Purchasing Power Parity (PPP) was 1.46 for Australia and 0.619 for the United Kingdom with the United States being 1. PPP represented the number of monetary units to buy the same representative basket of consumer goods and services [20].

## Competing interests

The authors declare that they have no competing interests.

## Authors’ contributions

DS led the study and conceived the original study idea. RS, RP and SK undertook the modelling, and EC generated the results. All authors provided expert input to the design of the study and the interpretation of the results. EC drafted the manuscript and all authors contributed to its editing and have read and approved the final submission.

## Pre-publication history

The pre-publication history for this paper can be accessed here:

http://www.biomedcentral.com/1471-2458/13/188/prepub
